# Quantitative relationships between elastic modulus of rod and biomechanical properties of transforaminal lumbar interbody fusion: a finite element analysis

**DOI:** 10.3389/fbioe.2024.1510597

**Published:** 2025-01-07

**Authors:** Jie Li, Zengfeng Du, Shuai Cao, Teng Lu, Zhongwei Sun, Hongyu Wei, Haopeng Li, Ting Zhang

**Affiliations:** ^1^ Department of Orthopedics, Second Affiliated Hospital of Xi’an Jiaotong University, Xi’an, Shaanxi, China; ^2^ Department of Orthopedics, The First Hospital of Yulin, Yulin, Shaanxi, China; ^3^ Department of Orthopedics, Civil Aviation General Hospital, Beijing, China; ^4^ Anhui Polytechnic University, School of Mechanical and Automotive Engineering, Wuhu, Anhui, China; ^5^ Department of Orthopaedics and Traumatology, Beijing Hospital of Traditional Chinese Medicine, Capital Medical University, Beijing, China

**Keywords:** transforaminal lumbar interbody fusion, connecting rod, elastic modulus, finite element analysis, biomechanical performance

## Abstract

**Background:**

Currently, some novel rods with lower elastic modulus have the potential as alternatives to traditional titanium alloy rods in lumbar fusion. However, how the elastic modulus of the rod (rod-E) influences the biomechanical performance of lumbar interbody fusion remains unclear. This study aimed to explore the quantitative relationships between rod-E and the biomechanical performance of transforaminal lumbar interbody fusion (TLIF).

**Methods:**

The intact finite element model of L1-S1 was constructed and validated. Then 12 TLIF models with rods of different elastic moduli (ranging from 1 GPa to 110 GPa with an interval of 10 GPa) were developed. The range of motion (ROM) of the fixed segment, mean strain of the bone graft, and maximum von Mises stresses on the cage, endplate, and posterior fixation system models were calculated. Finally, regression analysis was performed to establish functional relationships between rod-E and these indexes.

**Results:**

Increasing rod-E decreased ROM of the fixed segment, mean strain of the bone grafts, and peak stresses on the cage and endplate, while increasing peak stress on the screw-rod system. When rod-E increased from 1 GPa to 10 GPa, ROM decreased by 10.4%–39.4%. Further increasing rod-E from 10 GPa to 110 GPa resulted in a 9.3%–17.4% reduction in ROM. The peak stresses on the posterior fixation system showed a nonlinear increase as the rod-E increased from 1 GPa to 110 GPa under most loading conditions. The *R* ^2^ values for all fitting curves ranged from 0.76 to 1.00.

**Conclusion:**

The functional relationships between rod-E and the biomechanical properties of TLIF were constructed comprehensively. When the rod-E exceeds 10 GPa, further increases may not significantly improve stability, however, it may increase the risk of fixation failure. Therefore, a rod with an elastic modulus of approximately 10 GPa may provide optimal biomechanical properties for TLIF.

## 1 Introduction

Currently, lumbar interbody fusion (LIF) and pedicle screw fixation systems are widely used in the treatment of various lumbar degenerative conditions (Rathbone et al., 2023; Xu et al., 2023). The most commonly used material in fixation systems is titanium alloy (Ti-6Al-4V). Titanium alloy has excellent structural rigidity, biocompatibility, osseointegration properties, and corrosion resistance (Litak et al., 2022; Oda et al., 2022). Numerous clinical studies have confirmed its ability to provide robust stability and improve fusion rates (Qi et al., 2013; Kia et al., 2022). However, the elastic modulus of titanium alloy (110 GPa) is significantly higher than that of human bone tissue (0.1–30 GPa), which is referred to as ‘rigid fixation’ (Fan et al., 2021). The mismatch in elastic modulus is prone to stress concentration of the fixation and stress shielding of the interbody graft, which is closely associated with screw loosening, fusion failure, and possibly even revision surgery (Li W. et al., 2023; Li et al., 2018; Wu et al., 2023).

Ideally, a posterior pedicle screw fixation system should provide sufficient stability to promote fusion without excessive rigidity, thereby reducing instrumentation-related complications. To address the above issues, scholars have developed various connecting rods with lower elastic moduli to decrease the stiffness of the fixation system, such as polyetheretherketone (PEEK, 3.6 GPa) (Li J. et al., 2024; Li C. et al., 2024; Ponnappan et al., 2009), biodegradable rods (6.6 GPa) (Hsieh et al., 2020), ultra-high molecular weight polyethylene (UHMWPE, 11 GPa) (Biswas et al., 2018), carbon fiber-reinforced PEEK (CFRP, 18 GPa) (Biswas et al., 2022), and Nitinol rods (75 GPa) (Fan et al., 2019). To assess the biomechanical properties of these materials, researchers have conducted many studies based on numerical models or *ex vivo* specimens. The results consistently indicated that a reduction in the elastic modulus of rods (rod-E) can alleviate stress concentration in posterior instrumentation and mitigate the stress shielding effects in interbody bone grafts (Ahn et al., 2008; Cheers et al., 2024; Biswas et al., 2019). However, in both numerical model predictions and experimental studies, these low-elastic modulus rods have primarily been compared to titanium alloy rods on a one-to-one basis. Due to the inevitable heterogeneity among studies, a cross-comparison of these novel materials remains elusive. Therefore, previous research has only partially revealed the trend of how rod-E affects the biomechanics of lumbar fusion. To date, researchers have been unable to accurately predict the biomechanical response of lumbar fusion to rods with different elastic moduli, as a precise functional relationship or regression model between them has yet to be established.

Finite element analysis is a numerical simulation method that serves as an effective tool in studying the biomechanics of the lumbar spine (Xu et al., 2017). Compared to *in vivo* and *in vitro* experimental tests, finite element analysis offers lower costs and higher efficiency (Xu et al., 2024). More importantly, it can be used to determine the stress and strain that occur in each element of the structure when external forces are applied (Kim et al., 2022). These indexes are difficult to measure through experimental methods. This study aimed to explore the effect of rod-E on the biomechanical performance of transforaminal lumbar interbody fusion (TLIF) using finite element analysis and to determine a functional relationship between them. Once this quantitative relationship is established, researchers can quickly assess the biomechanical influence of rods with any elastic modulus within a specific range on TLIF, providing a convenient and rapid reference for future rod development and optimization.

To this end, we constructed 12 TLIF models with connecting rods of varying elastic moduli (ranging from 1 GPa to 110 GPa with an interval of 10 GPa). The range of motion (ROM) of the fixed segment, the mean strain of the bone graft, and the maximum stresses on the cage, endplate, and posterior fixation system were calculated in each model. Finally, we performed regression analysis to fit functional relationships between the rod-E and the biomechanical properties of TLIF.

## 2 Materials and methods

### 2.1 Reconstruction of the lumbar spine

**FIGURE 1:**
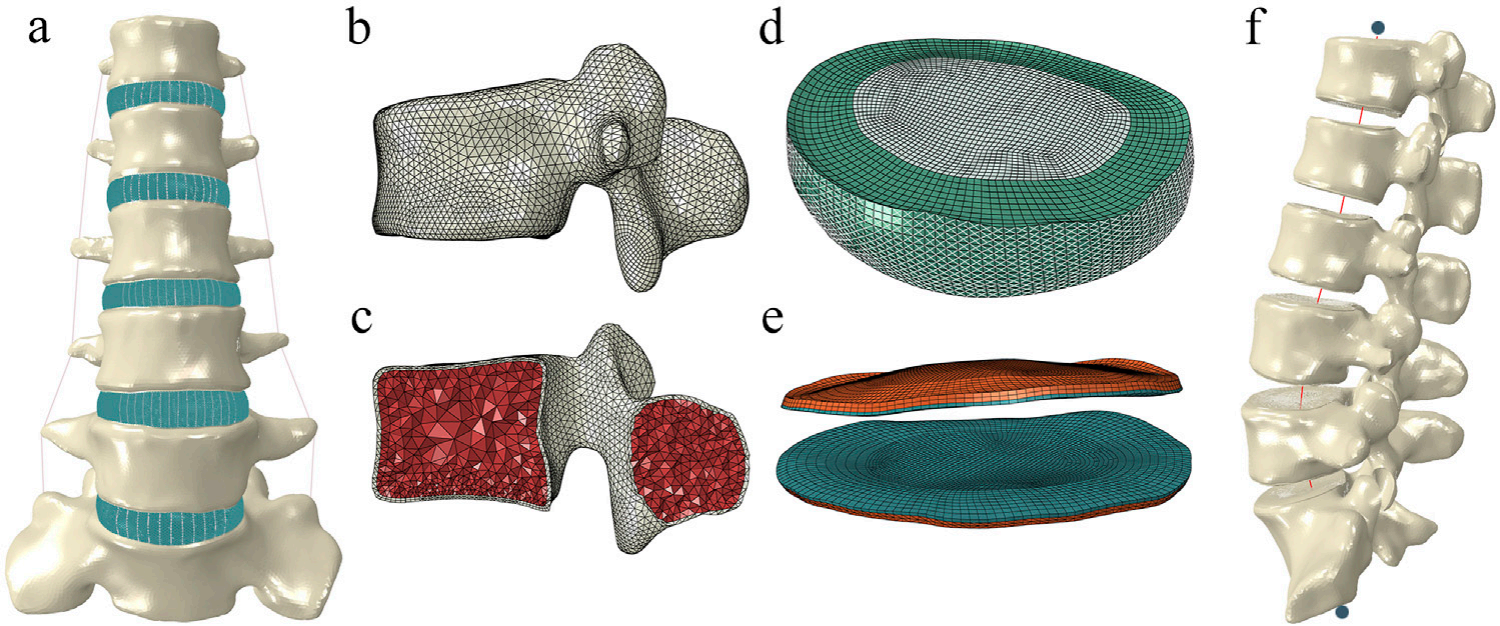
The intact finite element model of the human lumbar spine. **(A)** Front view of L1-S1 spine. **(B)** Lateral view of L1 vertebrae. **(C)** Longitudinal section of L1 vertebrae. **(D)** Intervertebral disc. **(E)** cortical and cartilage endplate. **(F)** Follower load path.

We first reconstructed an intact finite element model of the human lumbar spine (L1-S1), with the modeling process detailed in our previously published literature (Li J. et al., 2024; Li J. et al., 2023; Li K. et al., 2024). Briefly, computed tomography images of the lumbar spine from a healthy adult were imported into Mimic software (Materialise Inc., Leuven, Belgium) to reconstruct the vertebral geometric models. In 3-Matic application (Materialise Inc.), the cortical shell, defined by inwardly offsetting the vertebral surface by 1.0 mm, enclosed the inner region representing the cancellous bone. The intervertebral disc was also generated in 3-Matic through the Sweep-Loft operation. The construction of ligaments and collagen fibers, meshing tasks, and model assembly were implemented in the Hypermesh application (Altair Engineering Inc., Tory, Michigan, United States). Preprocessing, simulation, and postprocessing tasks were executed using the ABAQUS software package (Hibbitt, Karlsson, and Sorensen, Inc., Providence, Rhode Island United States). Figure 1 shows the final finite element model of the lumbar spine.

**FIGURE 2:**
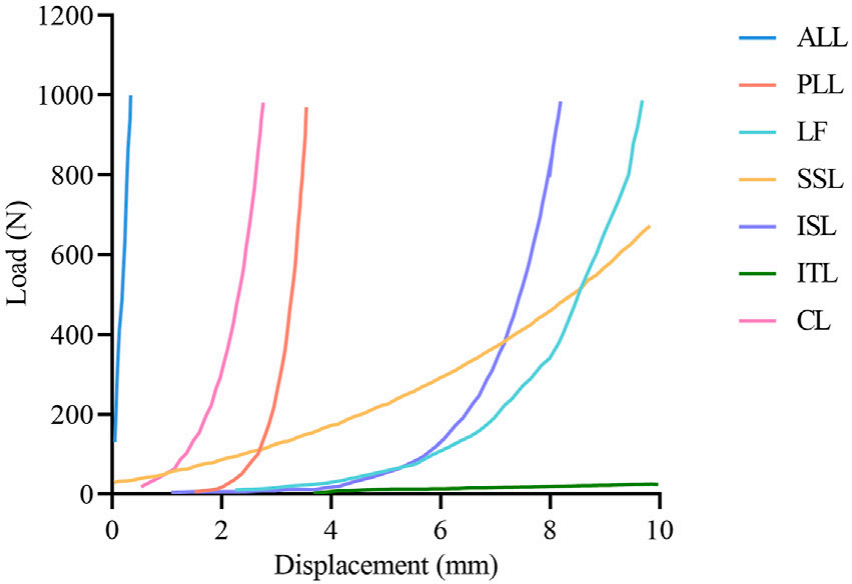
The load-displacement curve of ligaments in the finite element model. ALL, anterior longitudinal ligament; PLL, posterior longitudinal ligament; LF, ligamentum flavum; SSL, supraspinous ligament; ISL interspinous ligament; ITL, intertransverse ligament; CL, capsular ligament.

The thicknesses of the cortical bone, cortical endplate, and cartilage endplate were set to 1 mm, 1 mm, and 0.5 mm, respectively (Lu and Lu, 2020). A Python script was used to generate the collagen fibers of ten annulus fibrous layers, which are embedded within the annular matrix at approximately 30°–45° to the transverse plane (Xu et al., 2013). Seven types of ligaments were modeled using 2-node tension-only truss elements with nonlinear load-displacement responses (Figure 2). A soft contact algorithm with a friction coefficient of 0.1 was employed to simulate the contact behavior of the facet joints (Pradeep et al., 2024). The solid components were assembled by sharing nodes. A convergence analysis was conducted to limit the maximum changes in the strain energy to below 5% (Li K. et al., 2024; Umale et al., 2022). The skewness of all elements was maintained below 0.5. The aspect ratio was kept below 5 (Tsouknidas et al., 2013). Detailed element types, constitutive laws, and material parameters were referenced from previous studies, as shown in Table 1 (Lu et al., 2022; Sun et al., 2022; Lu et al., 2024).

**TABLE 1:** Materials properties and element types in the finite element models.

Materials	Element type	Constitutive law	Young’s modulus	Poisson’s ratio (μ)
Cortical bone	C3D4	Isotropic elastic	12, 000 MPa	0.3
Cancellous bone	C3D4	neo-Hookean	C_10_ = 19.38 MPa, D = 0.0252 MPa	
Bony endplate	C3D8I	Isotropic elastic	1, 200 MPa	0.29
Cartilage endplate	C3D8I	neo-Hookean	C_10_ = 4.10 MPa, D = 0.03 MPa	
Nucleus pulposus	C3D8H	Mooney-Rivlin	C_10_ = 0.12 MPa, C_01_ = 0.03 MPa	
Annulus ground	C3D8H	Mooney-Rivlin	C_10_ = 0.18 MPa, C_01_ = 0.045 MPa	
Annulus fiber	T3D2	Hypoelastic	360–550 MPa	
Screws	C3D4	Isotropic elastic	110,000 MPa	0.3
Rods	C3D4	Isotropic elastic	1 GPa, 10 GPa, 20 GPa, 30 GPa, 40 GPa, 50 GPa, 60 GPa, 70 GPa, 80 GPa, 90 GPa, 100 GPa, 110 GPa	0.3
Cage	C3D8	Isotropic elastic	3, 600 MPa	0.25
Bone grafts	C3D8	Isotropic elastic	100 MPa	0.2

### 2.2 Construction of the TLIF models

**FIGURE 3:**
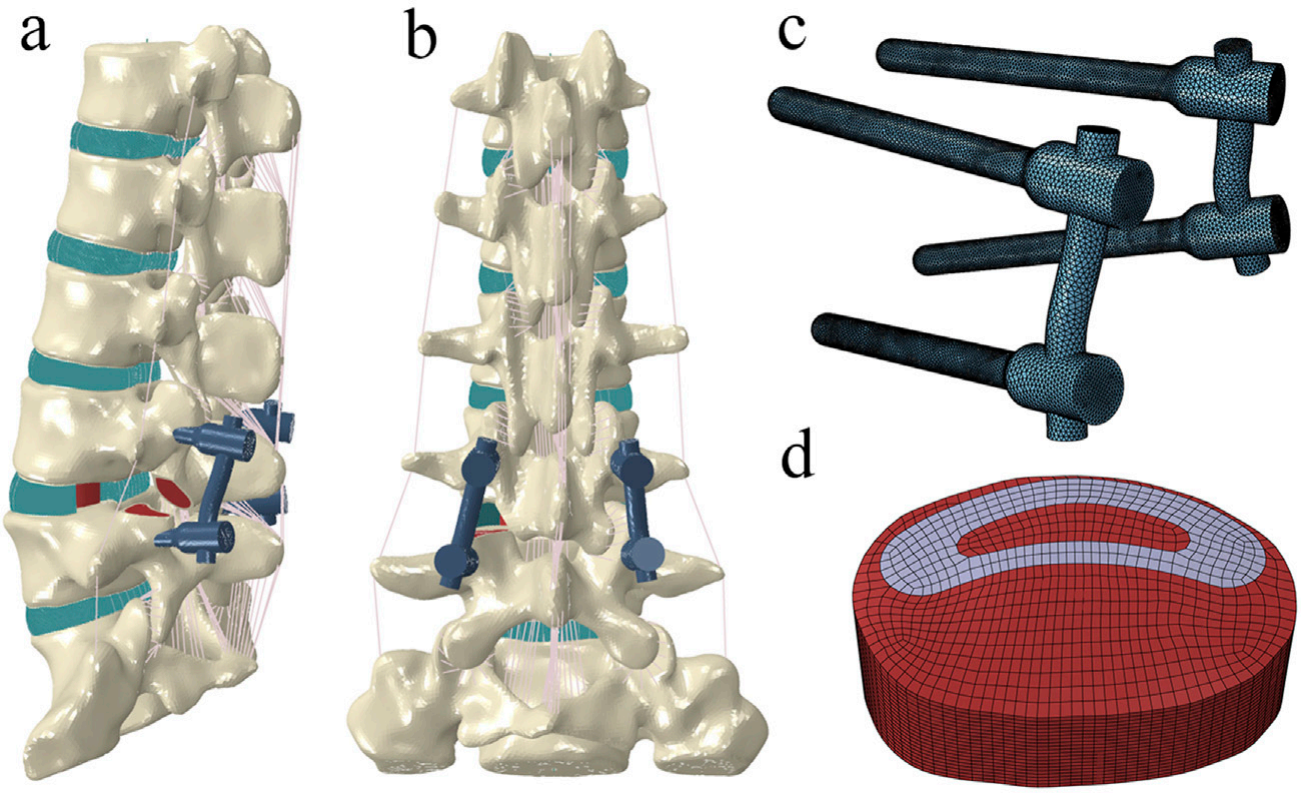
The surgical model for transforaminal lumbar interbody fusion. **(A)** left posterior oblique view. **(B)** posterior view. **(C)** pedicle screw–rod fixation. **(D)** cage and bone grafts.

Based on previous studies, the intact model was modified to simulate L4/5 TLIF with pedicle screw fixation (Liu et al., 2024). First, the left facet joint of the L4/5 segment was resected, followed by the removal of the left capsular ligament, part of the ligamentum flavum and annulus fibrosus, and the entire nucleus pulposus (Figure 3). After decompression, part of the cartilage endplate was removed to simulate the preparation of the bone graft bed. Subsequently, a cage (length = 30 mm, width = 10 mm, VERTE-STACK^®^ CRESCENTTM, Sofamor Danek, United States) was inserted into the intervertebral space, and the cage groove (central grafts) and the remaining space around the cage (peripheral grafts) was filled with bone grafts. Finally, a pedicle screw fixation system was appended. The diameter of the rod was 5.5 mm, and the diameter and length of the pedicle screw were 6.5 mm and 45 mm, respectively. The interfaces of the cage-endplate, cage-graft, graft-endplate, screw-bone, and screw-rod were set as fully bonded conditions via node sharing.

The types and material properties of the screws, rods, cage, and bone grafts are listed in Table 1. In this study, we developed 12 TLIF models, each with different elastic moduli assigned to the connecting rod (1 GPa, 10 GPa, 20 GPa, 30 GPa, 40 GPa, 50 GPa, 60 GPa, 70 GPa, 80 GPa, 90 GPa, 100 GPa, and 110 GPa).

### 2.3 Boundary and loading conditions

The inferior surface of the S1 vertebra in all models was fully constrained. This study adopted a hybrid loading protocol, incorporating follower load and moment load. Initially, a follower load of 400 N was applied to the intact model to simulate the upper body weight of a normal adult and the corresponding muscle forces. To achieve this, thermo-isotropic truss elements were created, and the desired follower load magnitude was generated by altering the temperature field of the truss elements (Sun et al., 2022). Regarding the load path, it was adjusted and optimized along the physiological curvature of the spine to ensure that the rotation produced by the follower load was less than 1° in each anatomic plane and each motion segment (Sun et al., 2022). Then, moment loads were applied in the intact model to simulate flexion, extension, lateral bending, and axial rotation of the spine. A reference point was created for the application of moments, located at the rotational center of L1, and coupled with the superior endplate of L1.

For the intact model, a 400 N follower load and an 8 N m moment were applied. The ROM at each segment was compared to values reported in the literature to validate the model (Panjabi et al., 1994; Shim et al., 2008). To further verify the model, the intradiscal pressure (IDP) at the L4/5 segment under pure follower loads was compared with literature data (Park et al., 2024; Dreischarf et al., 2014). For the surgical models, an initial follower load of 400 N was applied. Subsequently, the L1–S1 ROM derived from the intact model was imposed on the surgical models to ensure that their L1–S1 ROM matched that of the intact model.

### 2.4 Data extraction and regression analysis

For each surgical model, the following data was extracted: 1) ROMs of the fixed segment, 2) mean strain of the central and peripheral grafts, and 3) maximum von Mises stresses of the cage, endplate, screw, rod, and screw-bone interface. To establish the functional relationship between rod-E and the biomechanical properties of TLIF, the regression analysis was performed using GraphPad Prism 10 software (Boston, United States). In this study, we initially explored logarithmic interpolation. However, this approach did not fit the data well. To achieve a better curve fit, we subsequently adopted polynomial interpolation. The coefficient of determination (*R* ^2^) was used to indicate the goodness of fit.

## 3 Results

### 3.1 Model validation

**FIGURE 4:**
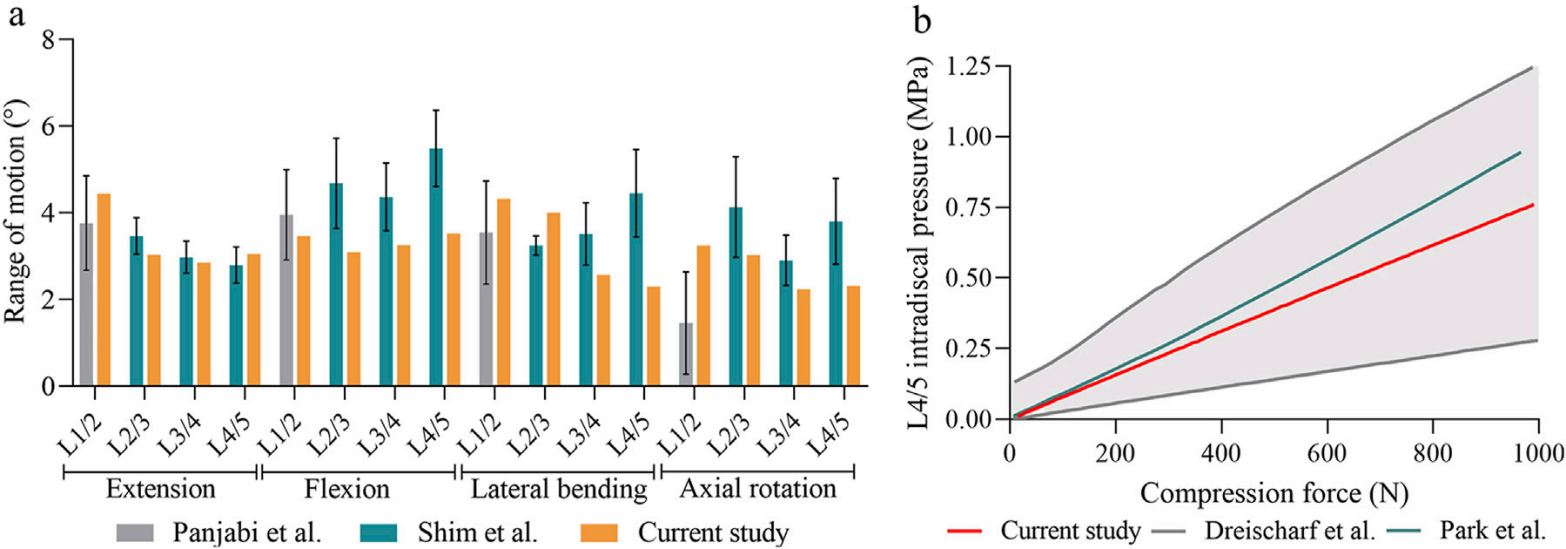
The validation of the intact model. **(A)** Comparison of the range of motion at each segment between the current and previous studies. **(B)** Comparison of intradiscal pressure at the L4–L5 segment in this study and those in previous studies.

To validate the reliability of the model, we first compared the ROM of each segment in the intact model with the results presented by Panjabi et al. (1994), Shim et al. (2008). Our findings indicated that, under similar loading conditions, the ROM values for most segments fell within the range reported in previous studies (Figure 4A). Additionally, we plotted the relationship between the IDP at the L4/5 segment and compressive load (Figure 4B). The results demonstrated a linear increase in IDP with rising compressive loads, consistent with previous reports, thereby further confirming the reliability of the model (Park et al., 2024; Dreischarf et al., 2014).

### 3.2 ROM of fixed segment

**FIGURE 5:**
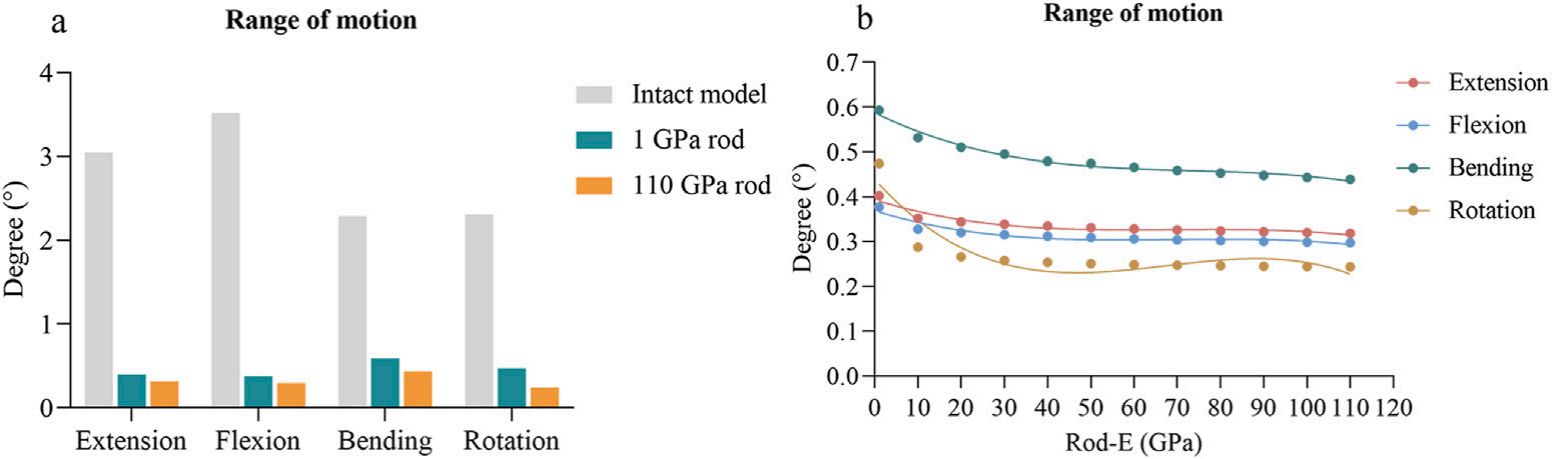
Changes of ROMs at the fixed segment in surgical models. **(A)** Comparison of the ROMs between the intact model and surgical models with 1 GPa rods and 110 GPa rods. **(B)** Changing trends and fitted curves of the ROMs as the rod-E increased from 1 GPa to 110 GPa. ROM, range of motion.

Compared to the intact model, the ROMs of the fixed segment in all surgical models were reduced by 87%–90% during extension, 89%–91% during flexion, 74%–81% during lateral bending, and 80%–90% during axial rotation (Figure 5A). The scatter plots were depicted to illustrate the change of ROM with rod-E (Figure 5B). The ROM exhibited a nonlinear decrease as the rod-E increased under all loading conditions. Notably, the ROM decreased by 10.4%–39.4% when the rod-E increased from 1 GPa to 10 GPa, especially during axial rotation. Once the rod-E exceeded 10 GPa, only a 9.3%–17.4% reduction in ROM was observed.

### 3.3 Mean strain of bone graft

**FIGURE 6:**
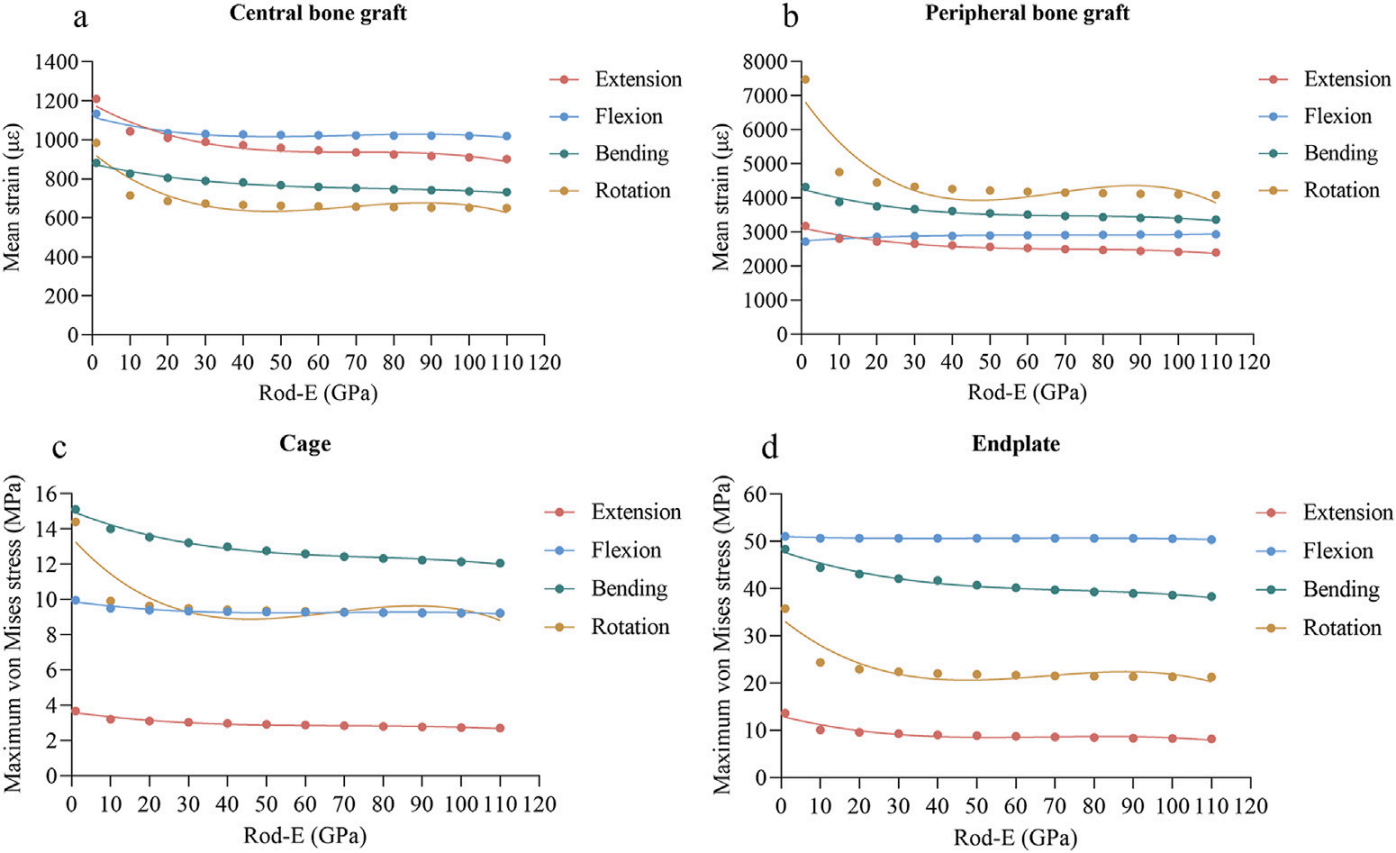
Changing trends and fitted curves of the mean strain of central grafts **(A)** and peripheral grafts **(B)** and peak stresses of the cage **(C)** and endplate **(D)** as the rod-E increased from 1 GPa to 110 GPa.

As the rod-E increased from 1 GPa to 110 GPa, the mean strain of the central grafts decreased from 1,209 με to 901 με during extension, from 1,134 με to 1,019 με during flexion, from 881 με to 732 με during lateral bending, and from 984 με to 650 με during axial rotation (Figure 6A). For the peripheral graft, the mean strain decreased from 3,179 με to 2,396 με during extension, increased from 2,716 με to 2,932 με during flexion, decreased from 4,326 με to 3,361 με during lateral bending, and decreased from 7,478 με to 4,091 με during axial rotation (Figure 6B).

### 3.4 Peak stress of cage and endplate

As the rod-E increased from 1 GPa to 110 GPa, the maximum von Mises stresses of the cage decreased from 3.68 MPa to 2.70 MPa during extension, from 9.97 MPa to 9.23 MPa during flexion, from 15.12 MPa to 12.07 MPa during lateral bending, and from 14.40 MPa to 9.21 MPa during axial rotation (Figure 6C). The maximum stresses of the endplate decreased from 13.64 MPa to 8.17 MPa during extension, from 51.09 MPa to 50.37 MPa during flexion, from 48.40 MPa to 38.34 MPa during lateral bending, and from 35.79 MPa to 21.26 MPa during axial rotation (Figure 6D).

### 3.5 Peak stress of pedicle screw fixation system

**FIGURE 7:**
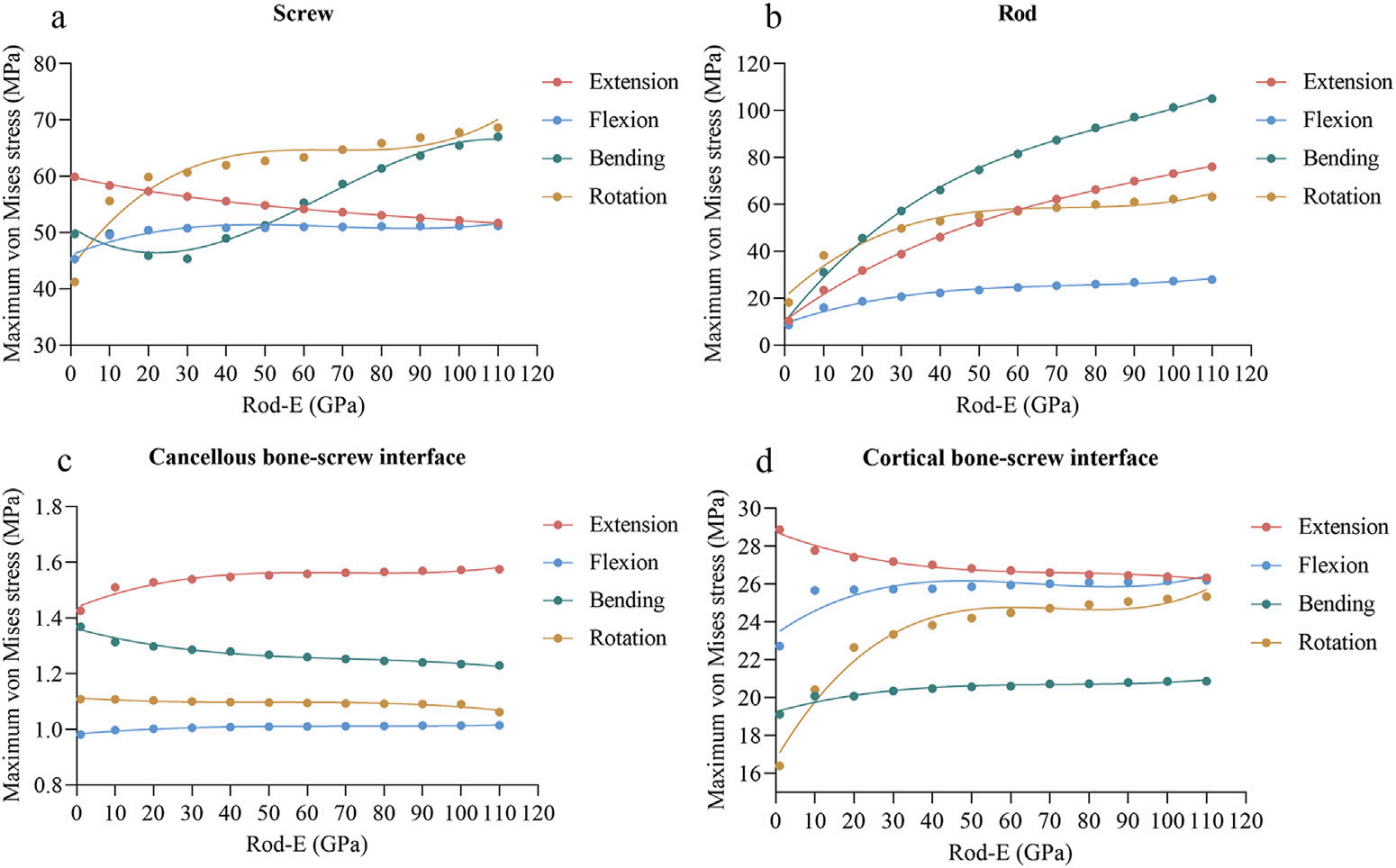
Changing trends and fitted curves of the peak stresses of the screws **(A)**, rods **(B)**, cancellous bone-screw interfaces **(C)**, and cortical bone-screw interfaces **(D)** as the rod-E increased from 1 GPa to 110 GPa.

As the rod-E increased, the maximum stresses on the screws decreased from 59.90 MPa to 51.70 MPa during extension, increased from 45.30 MPa to 51.20 MPa during flexion, and increased from 41.21 MPa to 68.65 MPa during axial rotation (Figure 7A). During lateral bending, the stress on the screws initially decreased but then gradually increased once the rod-E reached 30 GPa. For the rods, the maximum stresses increased from 10.44 MPa to 76.09 MPa during extension, from 8.52 MPa to 27.94 MPa during flexion, from 10.10 MPa to 105.03 MPa during lateral bending, and from 18.28 MPa to 63.21 MPa during axial rotation (Figure 7B). Regarding the cancellous bone-screw interface, the maximum stresses remained relatively stable across all loading conditions (Figure 7C). For the cortical bone-screw interface, the stresses increased to varying degrees in all conditions except extension, with the most pronounced rise occurring during axial rotation (Figure 7D).

### 3.6 Regression analysis

A polynomial regression analysis was performed to fit the functional relationship between rod-E and biomechanical properties of TLIF, including ROMs of the fixed segment, mean strain of the central and peripheral grafts, and maximum von Mises stresses of the cage, endplate, screw, rod, and screw-bone interface. The regression curves are those presented in Figures 5–7. In comparison to the other loading conditions, the curve fitting for axial rotation was less accurate. The regression equation is shown in Supplementary Table S1. The *R* ^2^ values for all fitting curves ranged from 0.76 to 1.00.

## 4 Discussion

The success of lumbar fusion surgery depends on the harmonious integration of the spine, internal fixation, interbody cages, and bone grafts (Brummund et al., 2017). The connecting rods are an essential component of the posterior pedicle screw fixation system, playing a critical role in load distribution during instrumented spinal fusion. Thus, the choice of rod material profoundly influences the biomechanical properties of the fusion. The elastic modulus of the rod (rod-E), which reflects its ability to resist deformation and recover its original shape, is a crucial parameter for evaluating the mechanical performance of the material (Biswas et al., 2019). However, how the rod-E affects the biomechanical behavior of lumbar fusion remains unclear. In this study, we investigated the effects of rod-E on the ROM of the fixed segment, the mean strain in the interbody bone grafts, and the peak stresses on the cage, endplate, screws, rods, and bone-screw interfaces. Finally, we used regression analysis to establish functional relationships between rod-E and these indexes. The finding indicated that a rod-E of approximately 10 GPa may provide the optimal biomechanical performance for TLIF. Our study provides preliminary insight into the quantitative relationship between rod-E and the biomechanical characteristics of TLIF, offering valuable reference data for the future development and improvement of rods.

The primary goal of lumbar fusion is to limit the motion of the fixed segments, based on the assumption that segmental instability is a cause of low back pain (Rohlmann et al., 2012). In this study, we evaluated the effect of rod-E on fixation stability. We used the ROM of the fixed segment as an indicator to represent fixation stability, as ROM has been widely adopted in finite element studies and experimental investigations to evaluate the stability of spinal fixation devices (Kim et al., 2022). Compared to the intact model, the ROM of the fixed segment was reduced by 74%–91% in all surgical models, which is consistent with the findings of Burkhard et al. (2023). They reported that pedicle screw instrumentation reduced lumbar segmental motion by approximately 80%. As the rod-E increased, the ROM decreased in all loading directions, indicating that even a rod with a modulus as low as 1 GPa can provide certain stability. Currently, PEEK, with an elastic modulus of 3.6 GPa, is the rod material with the lowest modulus reported in the literature. Studies have shown that PEEK rods can provide stability similar to that of titanium alloy rods, which aligns with the findings of this study Mavrogenis et al. (2014). Interestingly, we observed that increasing the rod-E from 1 GPa to 10 GPa reduced the ROM by 10.4%–39.4%, but further increasing it from 10 GPa to 110 GPa resulted in only a 9.3%–17.4% reduction in ROM. These results suggested that stability may be more sensitive to the rod material when the rod-E was below 10 GPa. However, when the rod-E exceeded 10 GPa, the stability showed no significant improvement. Therefore, from a stability standpoint, we hypothesize that using high-modulus materials like titanium alloys and other metals may be unnecessary, supporting the concept of semi-rigid fixation (Guan et al., 2023).

An ideal fixation system must achieve a balance between stability and load sharing. In this study, we also examined the effect of rod-E on load sharing. In theory, alterations in spinal load sharing directly affect the stress and strain distributions within specific structures (Ahn et al., 2008). Consequently, we employed the mean strain of bone graft and maximum von Mises stresses of the cage, endplate, and posterior fixation system to reflect load sharing of the spine. The von Mises stress reflects the state of stress within a material. It is a widely accepted index to evaluate whether a material might reach its yield strength and consequently fail (Fan et al., 2021; Fan et al., 2019). Mean strain is considered an appropriate indicator for assessing the risk of graft fusion failure, as low strain may inhibit bone fusion according to Wolff’s law (Li J. et al., 2024; Hsieh et al., 2020). Our results indicated that as the rod-E increased, the mean strain in both central and peripheral bone grafts, as well as the peak stresses on the cage and endplate, decreased to varying degrees under most loading conditions. Conversely, the peak stresses on the screws, rods, and bone-screw interfaces increased to varying extents under most loading conditions. The reason is that as the rod-E increases, the stiffness of the posterior column of the spine continuously rises, resulting in a gradual increase in the load borne by the posterior column and a corresponding decrease in the load carried by the anterior column (Qi et al., 2013). This load redistribution may subject the pedicle screw fixation system to excessive cyclic loading, leading to loosening or failure. Conversely, the cage and endplate in the anterior column experience reduced loading, thereby decreasing the risks of cage failure and endplate collapse. Additionally, the reduction in load on the intervertebral bone graft may increase the risk of fusion failure because the lack of mechanical stimulation is unfavorable for bone growth. Interestingly, we observed that the response of the screws during extension and lateral bending differs from that during flexion and axial rotation. Moreover, the peak stress of the cage under flexion and axial rotation appeared to overlap from 10 GPa onward. These phenomena may be attributed to the complex load-sharing mechanics and interplay among the screws, rods, cage, and lumbar spine. Unfortunately, the current study cannot fully elucidate the underlying reasons for the above results. Further research is needed to either confirm or challenge these findings.

Unlike the response of ROM to rod-E, the peak stresses on the screws, rods, and bone-screw interfaces showed a nonlinear increase as the rod-E increased from 1 GPa to 110 GPa under most loading conditions. This means that when the rod-E exceeds 10 GPa, further increases may not significantly improve stability, however, it may increase the risk of fixation failure. Therefore, we hypothesized that rods with an elasticity modulus of approximately 10 GPa may provide the optimal biomechanical performance for TLIF. The material reported in the literature that most closely matches this value is ultra-high molecular weight polyethylene (UHMWPE), which has an elastic modulus of 11 GPa (Biswas et al., 2018). However, the literature did not comprehensively reveal the biomechanical properties of this material when used as a connecting rod. It should be emphasized that considering both the maximum stress and the yield stress of a specific material is necessary. The ratio of maximum stress to yield stress serves as a useful indicator to evaluate the material performance (Fan et al., 2021). Overall, there is a need for future research to explore optimal rod material.

This study has several limitations. First, given the goals of our study, we simplified the model in many aspects, such as muscles, ligaments, and bones. Specifically, ligaments were modeled using 2-node tension-only truss elements instead of a solid ligament model which may provide greater accuracy (Baksiova et al., 2022). Additionally, muscle forces were simplified using follower loads, whereas a solid muscle model could offer a more physiologically accurate depiction (Kang et al., 2021). Moreover, we did not consider orthotropic bone properties, which can capture the direction-dependent mechanical characteristics (Baksiova et al., 2022). As such, the findings required further validation. Second, this study focused on single-segment TLIF, limiting the generalizability of the results. Third, although we established a functional relationship between rod-E and TLIF, our study only explored a modulus range of 1–110 GPa. Caution should be exercised when applying values outside this range. Finally, for all regression models, the first-order coefficients were significantly higher than the second and third-order coefficients, suggesting that the higher-order terms in the polynomial equations may lack practical significance.

## 5 Conclusion

This study investigated the effects of rod-E on the biomechanical properties of TLIF, including ROM of the fixed segment, the mean strain in the interbody bone grafts, and the peak stresses on the cage, endplate, screws, rods, and bone-screw interfaces. The functional relationships between rod-E and these indexes were established using regression analysis. The finding also indicated that rods with an elasticity modulus of approximately 10 GPa may provide the optimal biomechanical behavior for TLIF. These results provided a convenient and rapid reference for future rod development and optimization. More studies are needed to validate these results in the future.

## Data Availability

The original contributions presented in the study are included in the article/Supplementary Material, further inquiries can be directed to the corresponding authors.
