# Supplementary material for: Quantitative relationships between elastic modulus of rod and biomechanical properties of transforaminal lumbar interbody fusion: a finite element analysis
Source: Front Bioeng Biotechnol. 2025 Jan 7;12:1510597. doi: 10.3389/fbioe.2024.1510597 (PMC11752904; doi:10.3389/fbioe.2024.1510597)
Supplement: Supplementary file 1 [file Table1.docx]

Supplementary Material

**Supplementary Table S1**. Functional relationships between rod-E and biomechanical indexes of TLIF.

| Parameter | Regression equation | R^2^ |
| --- | --- | --- |
| ROM |  |  |
| Extension | Y=0.394 – 0.003*X + 4.532*10^-5^*X^2^ – 2.188*10^-7^*X^3^ | 0.915 |
| Flexion | Y=0.369 – 0.003*X + 4.520*10^-5^*X^2^ – 2.191*10^-7^*X^3^ | 0.905 |
| Bending | Y=0.587 – 0.005*X + 6.112*10^-5^*X^2^ – 2.777*10^-7^*X^3^ | 0.980 |
| Rotation | Y=0.439 – 0.011*X + 1.772*10^-4^*X^2^ – 8.745*10^-7^*X^3^ | 0.831 |
| Central bone graft (Mean strain, με) |  |  |
| Extension | Y=1182 – 10.54*X + 0.151*X^2^ – 7.225*10^-4^*X^3^ | 0.934 |
| Flexion | Y=1117 – 5.176*X + 0.084*X^2^ – 4.135*10^-4^*X^3^ | 0.832 |
| Bending | Y=875 – 4.220*X + 0.051*X^2^ – 2.247*10^-4^*X^3^ | 0.982 |
| Rotation | Y=933 – 15.53*X + 0.253*X^2^ – 1.251*10^-3^*X^3^ | 0.829 |
| Peripheral bone graft (Mean strain, με) |  |  |
| Extension | Y=3124 – 24.61*X + 0.334*X^2^ – 1.573*10^-3^*X^3^ | 0.955 |
| Flexion | Y=2733 + 7.614*X – 0.109*X^2^ + 5.176*10^-4^*X^3^ | 0.941 |
| Bending | Y=4266 – 31.07*X + 0.418*X^2^ – 1.937*10^-3^*X^3^ | 0.962 |
| Rotation | Y=6965 – 157.2*X + 2.564*X^2^ – 1.266*10^-2^*X^3^ | 0.830 |
| Cage (Stress peak, MPa) |  |  |
| Extension | Y=3.605 – 0.031*X + 4.216*10^-4^*X^2^ – 1.997*10^-6^*X^3^ | 0.951 |
| Flexion | Y=9.896 – 0.031*X + 4.722*10^-4^*X^2^ – 2.258*10^-6^*X^3^ | 0.915 |
| Bending | Y=15.02 – 0.088*X + 1.059*10^-3^*X^2^ – 4.606*10^-6^*X^3^ | 0.986 |
| Rotation | Y=13.51 – 0.244*X + 4.038*10^-3^*X^2^ – 2.010*10^-5^*X^3^ | 0.792 |
| Endplate (Stress peak, MPa) |  |  |
| Extension | Y=13.02 – 0.214*X + 3.266*10^-3^*X^2^ – 1.587*10^-5^*X^3^ | 0.894 |
| Flexion | Y=51.02 – 0.024*X + 4.826*10^-4^*X^2^ – 2.864*10^-6^*X^3^ | 0.882 |
| Bending | Y=47.94 – 0.286*X + 3.514*10^-3^*X^2^ – 1.575*10^-5^*X^3^ | 0.977 |
| Rotation | Y=33.70 – 0.672*X + 1.089*10^-2^*X^2^ – 5.357*10^-5^*X^3^ | 0.843 |
| Screw (Stress peak, MPa) |  |  |
| Extension | Y=59.91 – 0.146*X + 1.065*10^-3^*X^2^ – 3.794*10^-6^*X^3^ | 0.999 |
| Flexion | Y=46.01 + 0.274*X – 4.412*10^-3^*X^2^ +2.166*10^-5^*X^3^ | 0.877 |
| Bending | Y=51.02 – 0.447*X + 1.219*10^-2^*X^2^ – 6.217*10^-5^*X^3^ | 0.986 |
| Rotation | Y=43.59 + 0.954*X – 1.432*10^-2^*X^2^ + 7.124*10^-5^*X^3^ | 0.927 |
| Rod (Stress peak, MPa) |  |  |
| Extension | Y=10.48 + 1.202*X – 8.642*10^-3^*X^2^ + 2.878*10^-5^*X^3^ | 0.999 |
| Flexion | Y=9.26 + 0.574*X – 7.175*10^-3^*X^2^ + 3.222*10^-5^*X^3^ | 0.982 |
| Bending | Y=9.89 + 2.080*X – 1.896*10^-2^*X^2^ + 7.253*10^-5^*X^3^ | 0.999 |
| Rotation | Y=20.44 + 1.529*X – 2.075*10^-2^*X^2^ + 9.559*10^-5^*X^3^ | 0.972 |
| Cancellous bone-screw interface (Stress peak, MPa) |  |  |
| Extension | Y=1.439 + 5.542*10^-3^*X – 8.100*10^-5^*X^2^ + 3.859*10^-7^*X^3^ | 0.933 |
| Flexion | Y=0.983 + 1.160*10^-3^*X – 1.583*10^-5^*X^2^ + 7.230*10^-8^*X^3^ | 0.968 |
| Bending | Y=1.362 – 3.728*10^-3^*X + 4.610*10^-5^*X^2^ – 2.131*10^-7^*X^3^ | 0.972 |
| Rotation | Y=1.113 – 9.239*10^-4^*X + 1.857*10^-5^*X^2^ – 1.259*10^-7^*X^3^ | 0.876 |
| Cortical bone-screw interface (Stress peak, MPa) |  |  |
| Extension | Y=28.57 – 0.080*X + 1.028*10^-3^*X^2^ – 4.622*10^-6^*X^3^ | 0.973 |
| Flexion | Y=23.37 + 0.144*X – 2.357*10^-3^*X^2^ + 1.182*10^-5^*X^3^ | 0.764 |
| Bending | Y=19.26 + 0.057*X – 7.736*10^-4^*X^2^ + 3.572*10^-6^*X^3^ | 0.939 |
| Rotation | Y=16.74 + 0.351*X – 5.014*10^-3^*X^2^ + 2.334*10^-5^*X^3^ | 0.970 |
